# Protective Effect of Anti-Phosphatidylserine Antibody in a Guinea Pig Model of Advanced Hemorrhagic Arenavirus Infection

**DOI:** 10.2174/1874285801711010303

**Published:** 2017-10-30

**Authors:** John M. Thomas, Philip E. Thorpe

**Affiliations:** 1 The University of Texas Rio Grande Valley Department of Biology; School of Medicine 1201 W. University Drive, Edinburg, Texas 78539, USA; 2 The University of Texas Southwestern Medical Center Department of Pharmacology 2201 Inwood Road, Dallas, Texas 75390, USA

**Keywords:** Anti-phosphatidylserine, Hemorrhagic arenavirus infection, Phosphatidylserine, Pichinde virus, Cellular cytotoxicity

## Abstract

**Objective:**

Host derived markers on virally infected cells or virions may provide targets for the generation of antiviral agents. Recently, we identified phosphatidylserine (PS) as a host marker of virions and virally-infected cells.

**Methods and Materials:**

Under normal physiological conditions, PS is maintained on the inner leaflet of the plasma membrane facing the cytosol. Following viral infection, activation or pre-apoptotic changes cause PS to become externalized. We have previously shown that bavituximab, a chimeric human-mouse antibody that binds PS complexed with β2-glycoprotein I (β2GP1), protected rodents against lethal Pichinde virus and cytomegalovirus infections.

**Results:**

Here, we determined the antiviral activity of a fully human monoclonal antibody, PGN632, that directly binds to PS. Treatment with PGN632 protected 20% of guinea pigs with advanced infections of the hemorrhagic arenavirus, Pichinde, from death. Combining PGN632 with ribavirin improved the antiviral activity of both agents, such that the combination rescued 50% of animals from death.

**Conclusion:**

The major mechanisms of action of PGN632 appear to be opsonization of virus and antibody-dependent cellular cytotoxicity of virally-infected cells. PS-targeting agents may have utility in the treatment of viral diseases.

## 1 INTRODUCTION

Pichinde virus is a small, negative sense RNA virus among a diverse group of arenaviruses including Lassa virus, Junin virus, and Machupo virus capable of causing viral hemorrhagic fever (VHF) in humans [1, 2]. Due to the biodefense and public health risks associated with these viruses, the National Institutes for Health have classified these pathogens as category A agents. In spite of the threat posed by arenaviruses, no vaccines against them have been approved for human use, and the only approved antiviral agent is ribavirin [2]. While ribavirin has demonstrated efficacy in patients, concerns over toxicity in humans have limited its usage. In addition, ribavirin may drive viral evolution [3], giving rise to drug-resistant strains which further complicate viral clearance. It is therefore important to develop broad-spectrum antiviral agents that target the virus and virally-infected cells without contributing to viral resistance.

Bavituximab is a novel therapeutic antibody that has been shown to have activity [4] against the arenavirus, Pichinde virus, which causes a lethal disease in guinea pigs that closely models that caused by Lassa virus in humans [5, 28]. Bavituximab is a chimeric monoclonal antibody consisting of murine V_H_ and V_κ_ chains linked to human IgG1 constant domains. It targets a lipid, phosphatidylserine (PS), which is normally intracellular [6, 7], but becomes exposed on the outer membrane surface of virally-infected cells and on enveloped virions. Bavituximab binds with high affinity to PS complexed with the PS-binding plasma protein β2GP1 [8]. Administration of bavituximab to guinea pigs with advanced Pichinde virus infections enabled the animals to overcome their infections and recover [4]. Bavituximab acted by directly clearing opsonized virions from the bloodstream, and by eliminating virally-infected cells by antibody-dependent cellular cytotoxicity (ADCC). Bavituximab also bound to cells infected with multiple viruses, and rescued mice from lethal murine cytomegalovirus (mCMV) infection [4]. Pilot clinical trials in patients co-infected with chronic Hepatitis C virus and human immunodeficiency virus have shown that treatment with bavituximab appears well-tolerated with signs of reductions in virus load in the blood [9].

A series of fully human PS-targeting antibodies was generated by phage display technology to further explore the antiviral activity of PS-targeting antibodies. One of the antibodies, PGN632, was selected for its ability to bind directly to PS. An antibody that binds to PS without the requirement for β2GP1 has advantages in that it should not deplete β2GP1 from the blood, possibly giving a more robust antiviral effect not compounded by the clearance kinetics of β2GP1. Further, it is theoretically possible that an antibody such as PGN632 which does not require β2GP1-mediated interactions may be positioned closer to the viral envelope or surface of the cell, thus facilitating ADCC or interference with viral entry. The purpose of this study was to examine the efficacy and mode of action of PGN632 in the guinea pig Pichinde virus model.

## 2 MATERIALS AND METHODS

### 2.1 Antibodies

PGN632 and bavituximab were produced under serum free conditions by Avid Bioservices, a subsidiary of Peregrine Pharmaceuticals, Inc., Tustin, CA. Erbituximab (or Rituximab) was purchased from the UT Southwestern pharmacy, and is a chimeric human-mouse IgG1 monoclonal antibody which was used as a negative control. The PGN632 antibody was generated by Peregrine in collaboration with Affitech AG, Oslo, Norway. It is a fully human V_λ_ IgG1 that binds directly to PS with a K_D_ in the low nM range as determined by Biacore analyses. Bavituximab is a chimeric antibody consisting of mouse V_H_ and V_κ_ chains linked to human IgG1 constant domains and binds to human β2GP1:PS complexes with a K_D_ of 0.4nM.

### 2.2 Cells and Media

Vero-76 cells (CCL81; ATCC) were maintained in RPMI 1640 medium (Sigma-Alrdich), supplemented with antibiotics and 10% fetal bovine serum (Gibco). JH4 clone 1 (CCL158; ATCC) was the source of guinea pig fibroblasts used in the mechanistic studies. Fibroblasts were cultured in Ham's F12K medium (Gibco), supplemented with antibiotics and 10% fetal bovine serum (Gibco).

### 2.3 Viruses

Natural isolates of Pichinde virus are non-pathogenic in guinea pigs however, serially passaging Pichinde virus in guinea pigs has shown that more virulent strains can be generated which are lethal in the guinea pig model [5-28]. Using Pichinde virus CoAn 4763-P16 (generously provided by Dr. Judith Aronson at UTMB) as a starting inoculum, groups of Hartley guinea pigs (n=5) were inoculated intraperitoneally (i.p.) with 1 ml of virus. On day 6 post infection, spleens were harvested, pooled, and 10% (w/v) homogenates were prepared in RPMI 1640 media. These stocks were aliquoted and stored at -80C, and clarified by centrifugation before use. This initial Pichinde virus inoculum (P17) was then used as source material for another *in vivo* passaging experiment that generated a P18 isolate, and lastly we repeated the *in vivo* passaging one more time, thus generating a P19 isolate, termed Pichinde virus CoAn 4763-P19. Hereafter, when the term *Pichinde* virus is used within the context of our experimental studies, we are referring to the P19 isolate. This virus was quantitated by plaque assay on Vero cells, and used as our viral challenge stock for all *in vivo* and *in vitro* experiments. Viral titers of spleen stocks ranged between 10^6^ and 10^7^ plaque-forming units (PFU)/ml of homogenate.

### 2.4 Immunofluorescence Staining

Vero-76 cells were grown on chamber slides (BD Biosciences, San Jose, CA), and infected with Pichinde virus at a multiplicity of infection (MOI) of 5.0. At 48 hours post-infection, infected and uninfected cells were stained with PGN632 in the presence of β2GP1 at 37°C. Bavituximab and erbituximab were used as positive and negative controls, respectively. The cells were fixed in 4% paraformaldehyde, and incubated with anti-human FITC-conjugated antibodies (Jackson Immunoresearch, West Grove, PA). Cells were permeabilized with 0.1% Triton-X100. The cytoskeleton was stained with Texas red-phalloidin (Fisher Scientific Inc., Carlsbad, CA); nuclei were stained with Hoescht 33342 (Invitrogen, Grand Island, NY). Images were captured using a Coolsnap digital camera, and analyzed with MetaVue software (MDS Analytical Technologies Inc., Sunnyvale, CA).

### 2.5 Antibody Interactions with Virions or Virally-Infected Cells

For virus ELISAs, Immunolon U-bottom plates were coated with 100µl (approximately 10^6^ PFU) of Pichinde virus, and incubated at 4°C overnight. The next day, plates were washed and blocked with Superblock (Thermo Fisher Scientific Inc., Rockford, IL) supplemented with 1% BSA (Gibco). PGN632, bavituximab (positive control) and erbituximab (negative control) at 100µg/ml were mixed with 100µg/ml of human β2GP1 and serial ten-fold dilutions were performed along the plate. Binding was detected with an HRP-conjugated secondary antibody (Jackson Immunoresearch, West Grove, PA) and substrate O-phenylenediamine dihydrochloride (Sigma-Aldrich, St. Louis, MO). Absorbance was measured at 490nm.

To detect binding of PGN632 to Pichinde virus, Magprep anti-human IgG magnetic beads (Qiagen Inc., Valencia, CA) were coated with PGN632, bavituximab, or erbituximab. Streptavidin-coated Magprep beads (Qiagen Inc., Valencia, CA) were coated with biotinylated antibodies to guinea pig IgG and guinea pig antibodies to Pichinde virus in the presence of human β2GP1 (1µg/ml) at 37°C on a rotator for one hour. Next, we added 500 PFU of Pichinde virus to the beads, and incubated them for 30 minutes at room temperature. After this, beads and bound virus were removed using a magnet, and the remaining virus was quantified by plaque assay. The percentage removal of virus was calculated.

### 2.6 Phospholipid Specificity

Phospholipids (Avanti Polar Lipids, Inc.) were re-suspended in n-Hexane (Sigma-Aldrich, St. Louis, MO) and coated onto Immunolon U-bottom plates at 10µg/ml. Plates were washed, blocked and PGN632, in the presence or absence of β2GP1, was added to each plate at 100µg/ml. Serial ten-fold dilutions were performed, and goat-anti-human HRP-conjugated secondary antibody and substrate O-phenylenediamine dihydrochloride was used to detect bound PGN632. Reactions were stopped with 0.18M Na_2_HSO_4_, and absorbance was measured at 490nm [29].

### 2.7 Therapy Studies

Male Hartley guinea pigs (Charles River Laboratories International, Inc., Wilmington, MA) were infected i.p. with 10^5^ PFU of Pichinde virus isolate CoAn 4763-P19. This dose is equivalent to approximately 1000 lethal doses of virus. Animals were weighed and monitored daily for body temperature and physical appearance. Treatment was begun when signs of disease appeared, usually four days post-infection (animals had pyremia (>39ºC), had lost body weight and had disheveled fur). For single agent therapy, animals were treated i.p. with PGN632, bavituximab, or erbituximab at 6mg/kg per treatment, three times a week, for a maximum total of 12 doses. For combination therapy, animals were treated with antibody plus 3.25 mg/kg of ribavirin i.p. daily for a maximum of 10 doses

### 2.8 Virus Load Analyses

Guinea pigs were infected i.p. with a lethal dose of Pichinde virus. After the onset of disease signs (usually four days post-infection), animals were treated for one week with 6 mg/kg i/p. of PGN632, bavituximab, or erbituximab on days 7, 9, and 11. Groups of animals were sacrificed on days 10 and 14. Blood and major organs were collected and frozen. Tissues were homogenized and virus was quantified by plaque assay.

### 2.9 PT and aPTT Studies

To assess the effect of PGN632 on coagulation parameters, uninfected male guinea pigs were treated with 6 mg/kg i.p. of PGN632, bavituximab, or erbituximab. At 24 hours after treatment, citrated blood samples were collected. Prothrombin time (PT) and activated partial thromboplastin time (aPTT) were determined for recalcified blood samples by Antech Diagnostics (Fort Worth, TX).

### 2.10 Antibody-Mediated Cellular Ccytotoxicity

Guinea pig fibroblasts were used as target cells, and peritoneal exudate cells from thioglycollate-treated guinea pigs were used as effector cells. Fibroblasts were obtained from ATCC as described above, and peritoneal exudate cells were obtained by injecting guinea pigs i.p. with 15.0 ml of 3% thioglycollate medium (BD Difco). Four days after the injection, peritoneal exudate cells were harvested by aspirating the fluid from the peritoneal cavity. After removal of the contaminating tissue debris by cell strainer, red blood cells in the cell suspension were lysed with ammonium chloride solution. Cells were cultured in DMEM medium (Gibco), supplemented with 10% fetal bovine serum and antibiotics for 2 hours. Non-adherent cells were removed, and the adherent cells were collected and used for experiments. To measure antibody-mediated cellular cytotoxicity, fibroblasts were infected with Pichinde virus at an MOI of 5.0. PGN632, bavituximab, or erbituximab (20 µg/ml) in the presence of β2GP1 were added to the target cells 24 hours after infection. Effector cells were added to the cell cultures 48 hours after infection. Cytotoxicity was determined 18 hours later using a CytoTox 96 non-radioactive assay (Promega Corporation, Madison, MI) to determine cell vibility in antibody-treated versus untreated cell cultures.

### 2.11 Opsonization of virus

Guinea pigs were infected i.p. with 10^5^ PFU of Pichinde virus. After the onset of disease signs (usually four days post-infection), animals were treated with a single dose of PGN632, bavituximab, or erbituximab (6 mg/kg, i.p.). Animals were sacrificed 24 hours later, and whole blood was collected. The amount of virus present in the blood was quantified by plaque assay.

### 2.12 Virus-Specific Humoral Responses

Guinea pig sera from infected and treated animals were analyzed for the presence of virus-specific antibodies by ELISA. Immunolon U-bottom plates were coated with 100µl (approximately 10^6^ PFU) of Pichinde virus, and incubated at 4°C overnight. The next day, plates were washed and blocked with Superblock (Thermo Fisher Scientific Inc., Rockford, IL) supplemented with 1% BSA (Gibco). Plates were then incubated with guinea pig sera, and binding was detected with an HRP-conjugated goat anti-guinea pig antibody (Jackson Immunoresearch, West Grove, PA). Substrate O-phenylenediamine dihydrochloride (Sigma-Aldrich, St. Louis, MO) was added; absorbance was measured at 490nm.

### 2.13 Proliferative Responses to Viral Antigen

Antigen-specific immune responses were analyzed using a ^3^H-thymidine incorporation assay [4]. Briefly, animals were challenged i.p. with a lethal dose of Pichinde virus. After onset of disease signs at four days post-challenge, animals were treated with 4 doses of PGN632, bavituximab, or erbituximab (6 mg/kg, i.p.) given every 2 days for 8 days. Fourteen days after challenge, splenocytes were harvested and seeded at a density of 5 x 10^4^ cells per well in RPMI 1640 medium containing antibiotics and 10% FBS in round bottom 96-well plates (Thermo Fisher Scientific Inc., Rockford, IL). Splenocytes were stimulated with either extracellular viral antigen (10 µg/ml) obtained from multiple freeze-thawed viral stocks or mock antigen for 48 hours [4]. On day 3, cells were collected, washed, and pulsed with 1µCi per well of ^3^[H]-thymidine for eight hours (Amersham Biosciences Corporation, Piscataway, NJ). Stimulation index was calculated.

### 2.14 Statistical Analyses

The significance between two means (histograms) was calculated by student’s unpaired t-test. Log-rank analysis for non-normally distributed data (JMP SAS v12, Cary, NC) was used to compare the survival of the animals exposed to different treatments, and pairwise comparisons of treatment means were made between groups to determine statistical significance. Chi-square, degrees of freedom, and *p* values are reported. A value was considered significant if p ≤ 0.05.

### 2.15 Animal Usage

Male Hartley guinea pigs (Charles River Laboratories International, Inc., Wilmington, MA), aged 4-6 weeks, and approximately 300-350g in weight, were used for all animal studies. The animals were maintained in a specific pathogen free (SPF) BSL3 facility, and fed a normal diet of pig chow and water (unrestricted access to both). Prior to each animal experiment, guinea pigs were randomized and grouped, weighed, and sub-cutaneously implanted with biometric transponder chips that recorded identification number and temperature (Bio Medic Data Systems, Seaford, DE). Infected and/or antibody-treated animals were monitored twice daily for the entire duration of the experiment. Animals were euthanatized when their body weight decreased by 20% of their total body weight, or when they were scored as ‘severe’ based upon appearance, clinical signs, or unprovoked behavior in accordance with UT Southwestern Institutional Animal Care and Use Committee guidelines.

## 3 RESULTS

### 3.1 Recognition of PS is β2GP1 independent

**Fig. (1):**
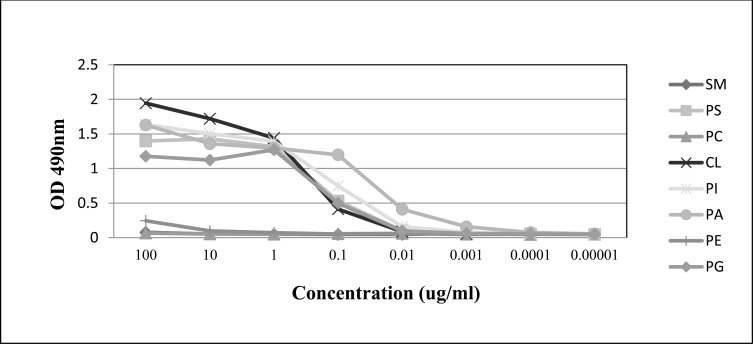
ELISA detection of PGN632 binding to immobilized PS. Antibody was added to ELISA plates coated with various phospholipids. Serial ten-fold dilutions of the antibodies were prepared. Plates were developed with HRP-labeled anti-human IgG followed by O-phenylenediamine dihydrochloride and the absorbance read at 490nm. PS, phosphatidylserine; CL, cardiolipin; PI, phosphatidylinositol; PA, phosphatidic acid; PG, phosphatidylglycerol; PC, phosphatidylcholine; PE, phosphatidylethanolamine; SM, sphingomyelin. Points; means of triplicate determinations.

PGN632 bound directly to PS-coated microplates. 50% maximal binding was observed with PGN632 at a concentration of 0.2 µg/ml (1.3nM) (Fig. **1**). Binding was not increased in the presence of human β2GP1 (not shown). Binding was also not due to carryover of trace amounts of contaminating β2GP1, which has confounded other studies [10, 11], since the antibodies were produced under serum free conditions. PGN632 bound directly to other anionic phospholipids including phosphatidylglycerol (PG), cardiolipin (CL), phosphatidylinositol (PI), and phosphatidic acid (PA) (Fig. **1**). PGN632 did not recognize any of the neutral phospholipids, sphingomyelin (SM), phosphatidylcholine (PC), or phosphatidylethanolamine (PE).

### 3.2 PGN632 Binds to Virally-Infected Cells

**Fig. (2):**
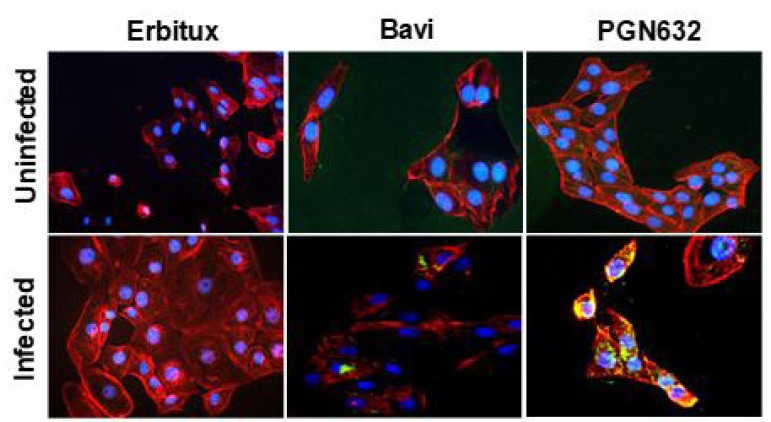
PGN632 binds to Pichinde virus-infected cells. Immunofluorescence staining of uninfected Vero cells (top panels) and Pichinde virus-infected cells (bottom panels) with antibody (green) 48h after infection. Cytoskeleton is stained red; nuclei are blue. Bavituximab/β2GP1 was used as a positive control and erbituximab as a negative control.

Immunofluorescence microscopy showed that PGN632 bound specifically to cells infected with Pichinde virus. Uninfected Vero cells were not visibly stained. After infection, the cells developed the same punctate staining pattern that we have previously observed with bavituximab on virally infected cells [4]. By 24-48 hours after infection, there was prominent staining of bleb-like structures on the surface of the cells, and weaker staining was visible over the entire cell surface (Fig. **2**).

### 3.3 PGN632 Binds to Virions

**Fig. (3):**
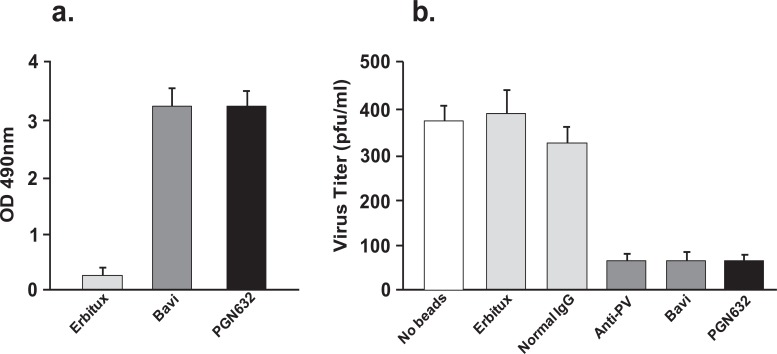
PGN632 binding to Pichinde virus. (***a***) ELISA: Pichinde virus was coated onto 96-well plates. Antibody (1μg/ml) was added in the presence of β2GP1 (1µg/ml) to enable binding of bavituximab. Columns, mean absorbance (n=3); error bars, S.E.M. **(b)** Depletion of infectious Pichinde virus by PGN632-coated magnetic beads. Beads coated with bavituximab or guinea pig antibodies to Pichinde virus (anti-PV) were used as positive controls. β2GP1 (1µg/ml) was added to enable binding of bavituximab. Beads coated with erbituximab or normal guinea pig IgG were used as negative controls. Columns, mean virus titer (n=3); bars, S.D.

PGN632 bound specifically to Pichinde virions adsorbed onto ELISA plates. The binding of the PGN632 was antigen-specific, since the erbituximab control antibody did not bind to the virions. PGN632 and bavituximab showed the same ability to bind to the immobilized virions (Fig. **3a**). To confirm that the PGN632 could bind to live infectious virions, bead depletion assays were performed in which magnetic beads coated with PGN632 were used to pull down infectious virus, and the number of infectious virus particles remaining in suspension were quantified by plaque assay. PGN632-coated beads removed 80% of the infectious virons, the same as for bavituximab-coated beads (Fig. **3b**). Erbituximab-coated beads did not remove the virions, showing that the depletion caused by the PGN632- and bavituximab-coated beads was antigen specific. A further control compared the depletion with that of beads coated with polyclonal guinea pig IgG purified from guinea pigs that had been immunized with Pichinde virus. The polyclonal anti-Pichinde virus-coated beads removed the same amount of virus from the suspensions as did the PGN632-coated beads (Fig. **3b**). Thus, PGN632 is capable of binding to PS on the external surface of the Pichinde virus envelope, at levels similar to those seen with bavituximab.

### 3.4 PGN632 is Well-Tolerated *in Vivo*

Uninfected guinea pigs treated with PGN632 (6 mg/kg, i.p., three times a week) gained body weight at the same rate as untreated animals. Their physical activity and appearance were indistinguishable from untreated animals. Because PGN632 binds to PS, it has the potential to be anti-coagulating through interference with coagulation factor binding to PS surfaces. We therefore measured PT and aPTT of blood drawn from uninfected guinea pigs 6 hours and 24 hours after treatment with PGN632 (6 mg/kg, i.p.). Neither PT nor aPTT in PGN632-treated animals was prolonged relative to erbituximab-treated or bavituximab-treated animals. (**data not shown**).

### 3.5 PGN632 Therapy Combined with Ribavirin Protects Guinea Pigs from Advanced Pichinde Virus Infection

**Fig. (4):**
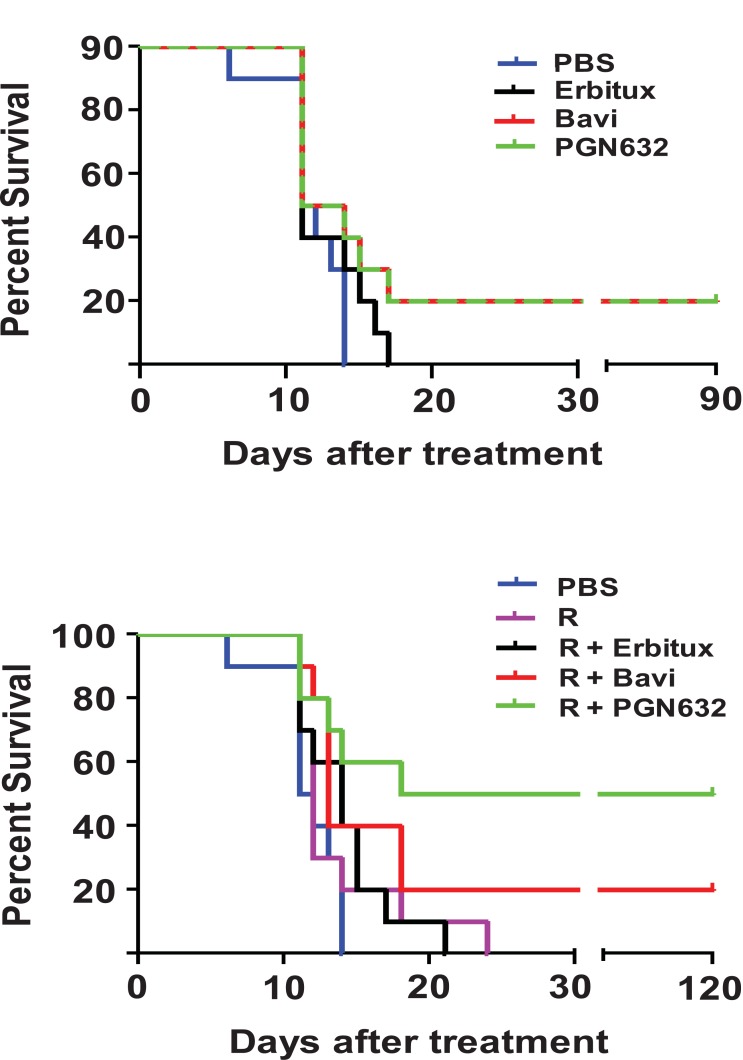
Survival of guinea pigs after lethal infection with Pichinde virus and PGN632 therapy and/or ribavirin. (**a**) Without ribavirin: guinea pigs (n=10) were infected with 10^5^ PFU of Pichinde virus (approx. 10^3^ lethal doses) and, starting 4 days later, were treated with PGN632 (6 mg/kg) i.p. three times a week for up to 3 weeks. X^2^ = 5.8543; degrees freedom = 3; p = 0.1189. **(b)** With ribavirin: the experiment was performed as in **(a)** except that the guinea pigs also received ribavirin (3.25 mg/kg, i.p.) daily for 10 days. Bavituximab was used as a positive control and erbituximab as a negative control. Surviving guinea pigs were re-challenged with 10^5^ PFU of Pichinde virus i.p. on day 60 **(a)** or day 90 **(b)** and were immune. The results are representative of two separate experiments. X^2^ = 14.6542; degrees freedom = 4; p = 0.0055.

Guinea pigs were infected with 10^5^ PFU (approximately 10^3^ lethal doses) of Pichinde virus, and treatment was begun when they showed signs of advanced infection (body temperature >39ºC, weight loss, disheveled appearance). When administered as a single agent, PGN632 protected 20% of guinea pigs from dying from their infections, the same percentage as for bavituximab (Fig. **4a**). While notable, the difference in survival between groups treated with anti-PS antibody and the PBS control animals was not significant (p = 0.1189). The guinea pigs started to regain body weight and gradually returned to having normal physical signs over the following 10-12 days. As expected, animals treated with erbituximab were not protected from challenge and did not survive past day 15.

We next determined if PGN632 offered better protection when given in combination with ribavirin, the primary drug for treating Lassa virus infection. Animals with advanced disease (as above) were treated with PGN632 three times a week in combination with daily ribavirin (3.25 mg/kg, i.p.) (Fig. **4b**). This sub-optimal dose of ribavirin offered no protection by itself. However, when combined with PGN632, 50% of guinea pigs recovered from their infections and survived, as compared to 20% in the group treated with PGN632 alone (Fig. **4b**). This difference trended towards, but did not reach, statistical significance (P=0.15). However, the difference in survival between the group treated with PGN632 and ribavirin, and the group treated with ribavirin alone was statistically-significant (p ≤ 0.0055). Thus, PGN632 appears to have additive anti-viral activity when combined with ribavirin.

### 3.6 Reduction of Viral Load in Tissues From Animals Treated With PGN632

**Fig. (5):**
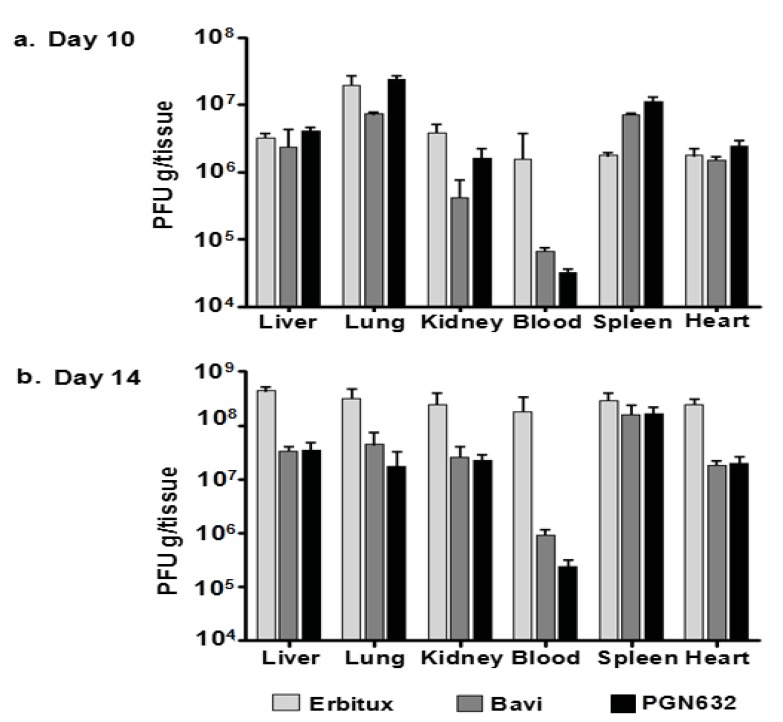
PGN632 treatment reduced the virus load in guinea pigs infected with Pichinde virus. (**a**) Virus load in tissues on day 10: Guinea pigs were infected with 10^5^ PFU of virus and were treated with PGN632 (6 mg/kg, i.p.) on day 4 and again on day 7. The guinea pigs were sacrificed on day 10. The histograms show virus load in PFU/g in the tissues. **(b)** Virus load in tissues on day 14: Guinea pigs were infected with 10^5^ PFU of virus and were treated with PGN632 (6 mg/kg, i.p.) on days 4, 7, and 10. They were sacrificed on day 14. The histograms show PFU/g in the tissues. Bavituximab treatment was given as a positive control, and erbituximab as a negative control. Columns, mean PFU per gram of tissue (n=3); bars, S.D.

Guinea pigs were treated with PGN632 on day 4 after infection, when they showed signs of advanced disease, and were treated again on day 7. Animals were sacrificed ten days post-infection, and the virus load in blood and various tissues was determined. The virus load in the blood was reduced by 50-fold relative to the group of animals that received erbituximab. However, there were no significant reductions of viral load in the liver, lungs, kidneys, or hearts of animals treated with PGN632 as compared to erbituximab recipients and the virus load in the spleen was elevated. Bavituximab recipients also showed no significant reductions in virus load in these tissues (Fig. **5a**). In contrast, in the group of animals sacrificed on day 14, after 3 treatments, there was a 500-fold reduction in virus load in the blood, and a 10-fold reduction in virus load in the liver, lung, kidney, and heart relative to erbituximab-treated animals (P<0.0001). Bavituximab also reduced the virus load in blood and tissues, but to a somewhat lesser extent than did PGN632 (Fig. **5b**). Virus load in the spleen was not reduced by either PGN632 or bavituximab treatment, probably because the spleen is a major site of clearance of opsonized virus from the blood.

### 3.7 PGN632 Causes Opsonization and Clearance of Pichinde Virus, and Induces Antibody-Dependent Cellular Cytotoxicity (ADCC) of Virus-Infected Cells

**Fig. (6):**
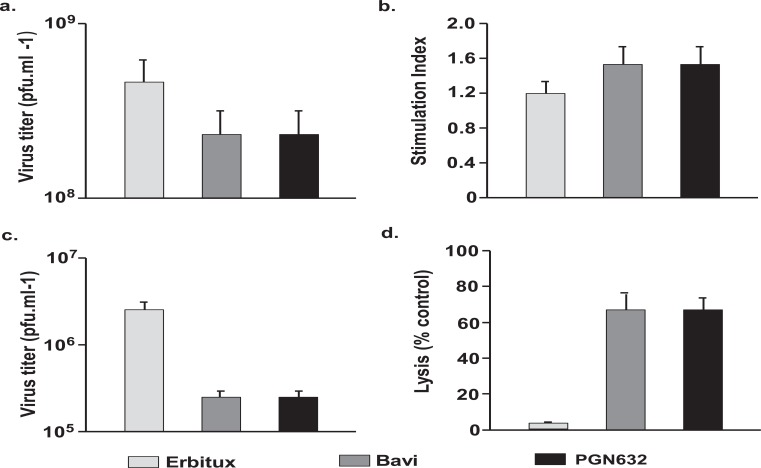
Mechanism of anti-viral effects of PGN632. (**a**) Lack of neutralization of virus. P388D1 cells were infected with Pichinde virus at an moi of 4 in the presence of antibody and β2GP1 (100µg/ml each). Cells and supernatants were collected on day 3, and virus quantified by plaque assay. Columns, average PFU/ml; bars, S.D. In all the above studies, bavituximab was used as a positive control and erbituximab as a negative control. PGN632 and bavituximab only reduce virus burden by 3-fold (not significant). **(b)** Significant lack of Pichinde virus antigen-specific proliferative responses. Splenocytes were collected from antibody-treated animals (n=3) 14 days after infection, and were stimulated in the presence of either virus or mock antigen, and their ability to incorporate 3[H]-thymidine was determined. Antibody therapy did not significantly increase the stimulation index. Columns, average stimulation index (n=3); bars, S.D. **(c)** Opsonization of Pichinde virus. Whole blood samples were collected from animals (n=3) that had been treated with antibody one day after infection, and virus quantified by plaque assay. Columns, average PFU/ml; bars, S.D. **(d)** ADCC of virus-infected cells. Primary guinea pig fibroblasts in tissue culture were infected with Pichinde virus and, 48 hrs later, were co-cultured with primary guinea pig peritoneal macrophages in the presence of PGN632. Lysis was determined by quantifying lactate dehydrogenase (LDH) release. PGN632 induced specific lysis of virus-infected cells. Columns, average percentages (n=3); bars, S.D.

We examined the mechanisms by which PGN632 rescued animals dying from Pichinde virus infections. First, we determined whether PGN632 was able to induce direct neutralization of Pichinde virus. Vero cells were infected with Pichinde virus (MOI = 4) in the presence of PGN632, bavituximab or erbituximab. PGN632 reduced the yield of virus only slightly (3-fold) and not significantly (Fig. **6a**). Bavituximab also reduced virus yield by 3-fold. Thus, neither PGN632 nor bavituximab are neutralizing antibodies.

Next, virus-specific humoral immune responses to Pichinde virus were assessed in guinea pigs that had been infected with virus 14 days earlier, and then treated with PGN632, bavituximab or erbituximab as above. The day 14 time point was selected because this is the time at which differences in survival became apparent. There were no significant differences in levels of anti-Pichinde virus antibodies in the PGN632 or bavituximab-treated animals compared to erbituximab treated animals (results not shown). Similarly, splenocytes taken from guinea pigs that had been sacrificed 14 days after infection and treated with PGN632 or bavituximab showed no significant proliferative responses to Pichinde antigens compared with splenocytes from erbituximab-treated guinea pigs (Fig. **6b**) Thus, induction of humoral or cellular immunity does not appear to explain the survival of PGN632-treated animals.

We determined whether antiviral activity could be due to opsonization of virus by PGN632 and subsequent clearance by the reticuloendothelial system. Guinea pigs were infected with Pichinde virus and treated i.p. with PGN632, bavituximab or erbituximab (6 mg/kg). Treatment with either PGN632 or bavituximab reduced the levels of circulating virus in the blood by 10 to 15-fold (P<0.01) relative to animals treated with erbituximab (Fig. **6c**). Thus, opsonization of virus may contribute to the better survival of PGN632-treated guinea pigs.

Lastly, we determined whether the antiviral activity of PGN632 could be due to ADCC of virus-infected cells. PGN632 and bavituximab efficiently mediated lysis of infected primary guinea pig fibroblasts by primary guinea pig macrophages *in vitro* whereas erbituximab did not (P < 0.0001). PGN632 and bavituximab were equally effective at mediating ADCC (Fig. **6a**) Thus, ADCC by innate immune cells likely contributed to the anti-viral action of PGN632.

## 4 DISCUSSION

The use of antibodies as novel therapeutics against infectious diseases has gained traction in recent years as an effective way to combat invasive pathogens. Synagis (palivizumab) is a humanized monoclonal antibody that has been approved for the treatment of respiratory syncytial virus [25]. Numerous other monoclonal antibodies are being developed for the treatment of a variety of viral diseases [12]. The PS-targeting chimeric antibody, bavituximab, has been tested in clinical trials in patients co-infected with hepatitis C virus and human immunodeficiency virus [9], and has demonstrated a capacity to bind other virally-infected cells and virions, including Ebola virus *in vitro* [27]. During the clinical trials, bavituximab therapy appeared safe and well tolerated, and reductions were observed in virus load in the blood. In the present study, we examined a new, fully human PS-targeting monoclonal antibody, PGN632, for antiviral activity in guinea pigs with advanced hemorrhagic arenavirus infections.

We recently identified PS as a generic marker of virus-infected cells and enveloped virions [4]. In uninfected cells, PS is sequestered to the inner leaflet of the plasma membrane under normal physiological conditions [7, 13]. Soon after virus infection (4 hours in Pichinde virus infected cells), PS becomes translocated to the outer surface of the cells [4]. Translocation could be due to activation of the cells by the virus and elevations of intracellular Ca^2+^ that activate PS exporters while simultaneously inhibiting the major PS importers, the aminophospholipid translocases [14, 15]. Alternatively, translocation could be related to pre-apoptotic changes [16]. Importantly, virions that egress from infected cells have exposed PS on their outer envelope surface. Acquisition of exposed PS appears to be a common feature of many enveloped viruses, including vesicular stomach virus [4], cytomegalovirus [4, 17], influenza [4], HIV-1 [18], vaccinia virus [19], and HSV [20]. Enveloped viruses that egress through the plasma membrane may acquire PS from raft-like structures which are enriched for PS [21]. Viruses that acquire their envelope from intracellular organelles, such as herpesviruses, may bud into vesicles that have not yet attained membrane asymmetry, or have lost lipid asymmetry as a consequence of the viral infection. Because anionic phospholipids on virus-infected cells are host-derived and independent of the viral genome, the acquisition of drug resistance could be less of a problem for PS-targeted antivirals than for agents that target virus-encoded components of the virus [24].

The PGN632 antibody has very similar phospholipid specificity to bavituximab, in that both antibodies recognize anionic phospholipids, including PS. Both anti-PS antibodies produced indistinguishable staining patterns on Pichinde virus-infected cells. PS is likely to be the major anionic phospholipid to become exposed on virus-infected cells since it is the most abundant, and it is the lipid whose asymmetry is most tightly regulated. However, it is possible that there is also exposure of the more-minor anionic phospholipids, including PI, PA and PG, which would also be detected by the antibodies. The major differences between PGN632 and bavituximab are: 1) PGN632 is human whereas bavituximab is chimeric and theoretically could induce antibodies against mouse determinants in patients and; 2) PGN632 binds to PS directly, without the need for the β2GP1 cofactor protein, which avoids possible depletion of β2GP1 in patients.

PGN632 treatment of guinea pigs with advanced Pichinde virus infections protected 20% of animals from death and reduced virus load in tissues. When combined with low-dose ribavirin, 50% of animals were protected, which would be expected for drugs with non-overlapping mechanisms of protection. We previously reported that 50% of guinea pigs could be protected from death by treatment with bavituximab alone, a higher percentage than has observed in the present studies. It is possible that these differences relate to the much higher virus inoculum used to infect the animals in the present study. It could also relate to the outbred nature of the guinea pig and genetic non-identity in the two studies. We chose to use the i.p. route for administration of the PGN632 antibody, as we had previously used this same route in our initial studies with bavituximab as an antiviral agent [4]. It is possible that better levels of protection could have been achieved if the antibody had been delivered using the intravenous (i.v.) route however, this is a time-intensive procedure that was beyond the scope of the current project. It has been shown that catheterized guinea pigs infected with Ebola virus are readily-available for blood draws and sample administration but, to date, no data exists that shows i.v. administration of anti-PS antibodies would achieve any improvement over i.p. injection within the context of arenavirus infections [27]. In humans infected with Lassa virus (another arenavirus that causes hemorrhagic fever), survival correlates with virus levels in the blood. Patients having virus levels below 10^6^ pfu/ml generally survive [1]. Indeed, clinical analysis of 137 samples of human patients infected with Lassa virus showed that, in the cases where Lassa virus infection was fatal, nearly all samples were viremic, with titers ranging from 10^3^-10^8^ TCID_50_/ml [26]. In the present studies, both PGN632 and bavituximab held the viremia in the guinea pigs below this level. Ribavirin is the only drug currently approved for treating human VHF, but is toxic when administered at doses that are considered to be efficacious, and it must be given early in infection to be effective [2, 22]. Combination with PGN632 or bavituximab may allow for reducing the dose of ribavirin to non-toxic levels, without sacrificing efficacy.

Two mechanisms appear to explain the antiviral activity of PGN632: (1) opsonization of virus by the antibody; and (2) the induction of antibody dependent cellular cytotoxicity (ADCC) of virally-infected cells [4]. PGN632 thus appears to act through the same mechanisms as bavituximab. A single dose of PGN632 reduced circulating Pichinde virus in the blood of infected guinea pigs by 10 to 15-fold. PGN632 also efficiently mediated the lysis of virally-infected guinea pig fibroblasts by primary guinea pig macrophages by ADCC. Together, these data suggest that protection is the result of limiting virus spread, either by direct clearance of virus from the blood, or by indirect clearance of virally infected cells in an antibody-dependent manner. PGN632, like bavituximab, was unable to neutralize infectivity of Pichinde virus. Thus, coating Pichinde virions with PGN632 does not obstruct virus entry. However, in an earlier study on HIV-1, we found that PGN632 inhibited CCR5-tropic primary HIV-1 isolates from infecting human peripheral blood mononuclear cells *in vitro*. The mechanism was that PGN632 induced β-chemokine production which blocked the R5 coreceptor [23]. Viral PS also is required for vaccinia virus infectivity [19]. Thus, it is possible that PS-targeting antibodies will have additional mechanisms of action with different viruses.

## CONCLUSION

In conclusion, using antibodies to target exposed PS on virus-infected cells and virions shows promise as an antiviral strategy. Because the antibodies act by different mechanisms, they should be used in combination with conventional antiviral agents. Other strategies for exploiting the exposure of PS on virus-infected cells and/or virus envelopes can be envisioned: antibodies, peptides or small molecules that target exposed anionic phospholipids might be used to deliver or direct agents that interfere with host cell or viral metabolism, or recruit immune effector cells.
